# Continuing Professional Development – Medical Imaging

**DOI:** 10.1002/jmrs.70112

**Published:** 2026-07-14

Maximise your continuing professional development (CPD) by reading the selected article and answering the five questions. Please remember to self‐claim your CPD and retain your supporting evidence. Answers will be available via the QR code and published in JMRS – Volume 73, Issue 4, December 2026.

## Diagnostic Performance of Ultrasound in Adult Appendicitis. A Retrospective Review of 331 Cases

Alistair Lock, Martin Necas. https://doi.org/10.1002/jmrs.70079

1. Which pathology was identified as the most common cause of right iliac fossa pain in women referred for ultrasound in this study?
  - Acute appendicitis
  - Ovarian cyst accident
  - Ureteric stone
  - Ectopic pregnancy
2. Based on the study findings, which statement best describes the ultrasound diagnoses identified in patients presenting with right iliac fossa pain?
  - Appendicitis was the most frequently identified diagnosis.
  - Appendicitis and alternative diagnoses were identified at similar rates.
  - Alternative diagnoses were identified approximately twice as often as appendicitis.
  - Alternative diagnoses were identified approximately three times as often as appendicitis.
3. What was the reported sensitivity of ultrasound for the detection of appendicitis in this study?
  - 50%
  - 55%
  - 60%
  - 70%
4. A patient undergoes ultrasound for suspected appendicitis, but the appendix is not visualised. Based on the methodology used in this study, how would this examination be classified for statistical analysis?
  - Positive for appendicitis
  - Negative for appendicitis
  - Excluded from analysis
  - Reclassified after CT correlation
5. Why might some published studies report appendicitis detection rates of 90% or higher with ultrasound?
  - Greater operator experience
  - More advanced ultrasound equipment
  - Higher pre‐test probability of appendicitis
  - Exclusion of patients in whom the appendix was not visualised

### Recommended Further Reading

1. D. Moris, E. K. Paulson, and T. N. Pappas. 2021. “Diagnosis and Management of Acute Appendicitis in Adults: A Review.” *JAMA* 326 (22): 2299–2311. https://doi.org/10.1001/jama.2021.20502.

2. J. Fu, X. Zhou, L. Chen, and S. Lu. 2021. “Abdominal Ultrasound and Its Diagnostic Accuracy in Diagnosing Acute Appendicitis: A Meta‐Analysis.” *Frontiers in Surgery* 8. https://doi.org/10.3389/fsurg.2021.707160.

3. F. Yuan, and M. Necas. 2015. “Retrospective Audit of Patients Presenting for Ultrasound with Suspicion of Appendicitis.” *Australasian Journal of Ultrasound in Medicine* 18 (2): 67–69. https://doi.org/10.1002/j.2205‐0140.2015.tb00044.x.

## Answers


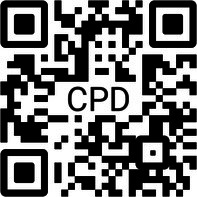
Scan this QR code or https://www.surveymonkey.com/r/JMRS_Sept2026_MI to find the answers

